# CsrA-Mediated Translational Activation of *ymdA* Expression in Escherichia coli

**DOI:** 10.1128/mBio.00849-20

**Published:** 2020-09-15

**Authors:** Andrew Renda, Stephanie Poly, Ying-Jung Lai, Archana Pannuri, Helen Yakhnin, Anastasia H. Potts, Philip C. Bevilacqua, Tony Romeo, Paul Babitzke

**Affiliations:** a Department of Biochemistry and Molecular Biology, The Pennsylvania State University, University Park, Pennsylvania, USA; b Center for RNA Molecular Biology, The Pennsylvania State University, University Park, Pennsylvania, USA; c Department of Microbiology and Cell Science, Institute of Food and Agricultural Sciences, University of Florida, Gainesville, Florida, USA; d Department of Chemistry, The Pennsylvania State University, University Park, Pennsylvania, USA; Institut Pasteur

**Keywords:** CsrA, RNA binding proteins, biofilms, gene regulation, translational control

## Abstract

The sequence-specific RNA-binding protein CsrA is the central component of the conserved global regulatory Csr system. In Escherichia coli, CsrA regulates many cellular processes, including biofilm formation, motility, carbon metabolism, iron homeostasis, and stress responses. Such regulation often involves translational repression by CsrA binding to an mRNA target, thereby inhibiting ribosome binding. While CsrA also extensively activates gene expression, no detailed mechanism for CsrA-mediated translational activation has been demonstrated. An integrated transcriptomic study identified *ymdA* as having the strongest CsrA-mediated activation across the E. coli transcriptome. Here, we determined that CsrA activates *ymdA* expression posttranscriptionally. Gel mobility shift, footprint, toeprint, and *in vitro* coupled transcription-translation assays identified two CsrA binding sites in the leader region of the *ymdA* transcript that are critical for translational activation. Reporter fusion assays confirmed that CsrA activates *ymdA* expression at the posttranscriptional level *in vivo*. Furthermore, loss of binding at either of the two CsrA binding sites abolished CsrA-dependent activation. mRNA half-life studies revealed that CsrA also contributes to stabilization of *ymdA* mRNA. RNA structure prediction revealed an RNA hairpin upstream of the *ymdA* start codon that sequesters the Shine-Dalgarno (SD) sequence, which would inhibit ribosome binding. This hairpin also contains one of the two critical CsrA binding sites, with the other site located just upstream. Our results demonstrate that bound CsrA destabilizes the SD-sequestering hairpin such that the ribosome can bind and initiate translation. Since YmdA represses biofilm formation, CsrA-mediated activation of *ymdA* expression may repress biofilm formation under certain conditions.

## Abstract (precis)

The Csr system of E. coli controls gene expression and physiology on a global scale. CsrA protein, the central component of this system, represses translation initiation of numerous genes by binding to target transcripts, thereby competing with ribosome binding. Variations of this mechanism are so common that CsrA is sometimes called a translational repressor. Although CsrA-mediated activation mechanisms have been elucidated in which bound CsrA inhibits RNA degradation, no translation activation mechanism has been defined. Here, we demonstrate that CsrA binding to two sites in the 5′ untranslated leader of *ymdA* mRNA activates translation by destabilizing a structure that otherwise prevents ribosome binding. The extensive role of CsrA in activating gene expression suggests the common occurrence of similar activation mechanisms.

## INTRODUCTION

Bacteria have evolved interconnecting global regulatory networks that assist in adaptation to changing environments and other stresses. One of these networks is the carbon storage regulatory (Csr) system in Escherichia coli. Conserved across many bacterial species, this network regulates expression of genes involved in carbon metabolism, iron homeostasis, motility, biofilm formation, the stringent response, cyclic-di-GMP synthesis, quorum sensing, and many other processes for survival and virulence (1–3). Recent integrated transcriptomic studies demonstrated that the Csr system controls expression of hundreds of genes in E. coli including numerous regulatory and stress response pathways (4–6). Furthermore, in both E. coli and *Salmonella*, such studies have suggested that while CsrA predominantly plays a role in repression of gene expression, it also extensively activates gene expression (6, 7). The underlying molecular mechanisms of how CsrA activates gene expression are largely unknown.

CsrA is a homodimeric protein with two symmetrical RNA-binding surfaces capable of binding to two sites within the same target RNA (8, 9). A systematic evolution of ligands by exponential enrichment (SELEX)-derived consensus sequence for CsrA binding (RUACARGGAUGU) contains a conserved GGA motif that is typically presented in the loop of a short hairpin (10), which is also characteristic of CsrA RNA binding sites identified *in vivo* (6). CsrA activity is regulated by two small RNA (sRNA) antagonists, CsrB and CsrC, which contain up to 22 and 13 CsrA binding sites, respectively. The high-affinity interaction of CsrA with these sRNAs outcompetes lower-affinity binding to target mRNAs by sequestering free CsrA dimers (11, 12).

The BarA-UvrY two-component signal transduction system activates transcription of CsrB and CsrC in response to short-chain carboxylic acids such as formate and acetate (13–15). Turnover of CsrB/C requires the membrane-bound GGDEF-EAL domain protein CsrD to initiate degradation by RNase E (16, 17). In the presence of glucose, CsrD binds to unphosphorylated, glucose-specific enzyme IIA (EIIA^Glc^) stimulating the decay of CsrB/C (18), while the absence of glucose results in stabilization of CsrB/C, elevation of CsrB/C RNA levels, and sequestration of free CsrA. Finally, CsrA regulates its own expression both by translational repression and indirect transcriptional activation, thereby tightly controlling the amount of free CsrA in the cell (19).

CsrA is known to repress translation of many genes by a variety of mechanisms that are dependent on the location of CsrA binding sites within the mRNA. The most commonly characterized mechanism involves CsrA binding to a site overlapping the Shine-Dalgarno (SD) sequence and/or translation initiation region, which competes with 30S ribosomal subunit binding (1, 2, 19–21). As this mechanism has been identified frequently, the notion that CsrA functions solely as a translational repressor has been emphasized. However, CsrA also participates in Rho-dependent termination by destabilizing an RNA secondary structure in the *pgaABCD* leader transcript that would otherwise sequester a Rho utilization (*rut*) site (22). CsrA is also known to activate *flhDC* expression, which encodes a DNA-binding regulatory protein required for flagellum biosynthesis (23). In this case, CsrA binding to the 5′ end of the *flhDC* transcript stabilizes this mRNA by preventing degradation via the 5′-end-dependent activity of RNase E (24). CsrA binding to CsrB also prevents cleavage of this RNA by RNase E. Consequently, CsrB decay requires a functional CsrD protein in addition to RNase E (17). There are also examples in which mRNA binding by CsrA appears to activate translation, although the underlying molecular mechanisms were not established (25, 26).

On the basis of an integrated transcriptomic study combining transcriptome sequencing (RNA-seq), ribosome profiling, and high-throughput sequencing of RNA isolated by cross-linking immunoprecipitation (CLIP-seq), we identified *ymdA* as having the strongest CsrA-mediated activation across the entire E. coli transcriptome in mid-exponential phase (6). Compared to the *csrA* mutant strain, the wild-type (WT) strain had a 9-fold-higher abundance of *ymdA* mRNA and a 27-fold increase in the number of ribosome protected fragments across the mRNA. Moreover, CLIP-seq experiments showed that CsrA cross-links to the leader region of *ymdA* mRNA. The function of YmdA is uncharacterized, although a previous study reported that overexpression of *ymdA* inhibited biofilm formation ∼5-fold and had a 2-fold-higher susceptibility to the aminoglycoside antibiotic apramycin (27). A *ymdA* knockout strain had no significant effect on biofilm formation, but it exhibited twofold increased resistance to apramycin (27).

In this study, we determined that CsrA activates *ymdA* translation by binding to two sites in the *ymdA* leader RNA, one of which is present in a structure that sequesters the SD sequence. We show that upon CsrA binding, this hairpin is destabilized, and the SD sequence becomes single stranded and available for ribosome binding. These findings provide direct evidence for CsrA-mediated translational activation of *ymdA* expression.

## RESULTS

### CsrA binds to two sites in the *ymdA* leader transcript causing destabilization of a SD-sequestering hairpin.

**FIG 1:**
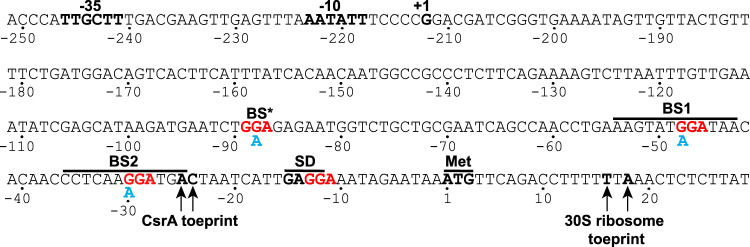
*ymdA* promoter and leader region. The −35 and −10 promoter elements, transcription start site (+1), Shine-Dalgarno (SD) sequence, and translation initiation codon (Met) are labeled. The GGA motifs of four potential CsrA binding sites are in red. CsrA binding sites 1 (BS1) and 2 (BS2) were identified as authentic binding sites, whereas BS* and the site overlapping the SD sequence were excluded as CsrA binding sites. The positions of CsrA and 30S ribosomal subunit toeprints are marked. Nucleotide substitutions in BS*, BS1, and BS2 are shown in cyan. Numbering is with respect to the start of *ymdA* translation.

**FIG 2:**
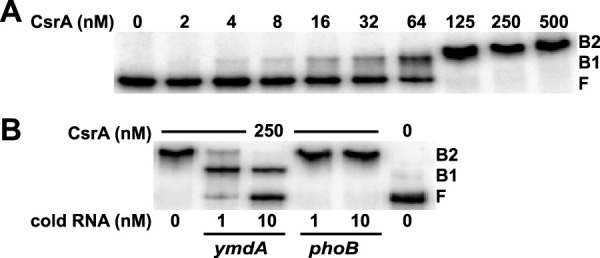
CsrA binds to *ymdA* leader RNA. (A) 5′-end-labeled *ymdA* RNA (0.1 nM) was incubated with the concentration of CsrA shown at the top of each lane. Positions of bound (B1 and B2) and free (F) RNA are labeled. The experiment was performed three times, and a representative gel is shown. (B) RNA competition assay to demonstrate the specificity of CsrA-*ymdA* RNA interaction. Labeled *ymdA* leader RNA (0.1 nM) was challenged with either specific (*ymdA*) or nonspecific (*phoB*) RNA competitors. The concentration of CsrA is shown above each lane. The concentration of unlabeled RNA is indicated below each lane. The positions of bound (B1 and B2) and free (F) RNA are labeled. The experiment was performed twice, and a representative gel is shown.

Previous CLIP-seq results indicated that CsrA binds to the leader region of the *ymdA* transcript (6). Four GGA motifs were identified within 90 nucleotides (nt) of the translation initiation codon; GGA is a critical component of a CsrA binding site (Fig. 1). Quantitative gel mobility shift assays were performed to investigate the interaction of CsrA with *ymdA* leader RNA containing all four GGA motifs (−100 to −1 relative to the start of *ymdA* translation). A distinct band indicative of bound CsrA was observed between 8 and 64 nM CsrA, with a second shift appearing at 125 nM CsrA and above (Fig. 2A). A nonlinear least-squares analysis of this data yielded a *K_d_* (dissociation constant) value of 48 ± 3 nM CsrA, indicating that CsrA binds to the *ymdA* transcript with moderate affinity. While the stoichiometry of each bound state was not investigated, it is likely that the first bound state (B1) is a single CsrA dimer bound to a single transcript, while the second bound state (B2) may contain two CsrA dimers bound to the same transcript. The specificity of this interaction was assessed by competitive gel mobility shift assays in the presence of specific (*ymdA*) or nonspecific (*phoB*) unlabeled RNA competitors in 10- and 100-fold excess to the radiolabeled *ymdA* transcript. With a CsrA concentration of 250 nM, unlabeled *ymdA* RNA competed for binding of CsrA to the radiolabeled *ymdA* transcript, whereas the nonspecific competitor did not, indicating that the interaction of CsrA with the *ymdA* RNA is specific (Fig. 2B).

**FIG 3:**
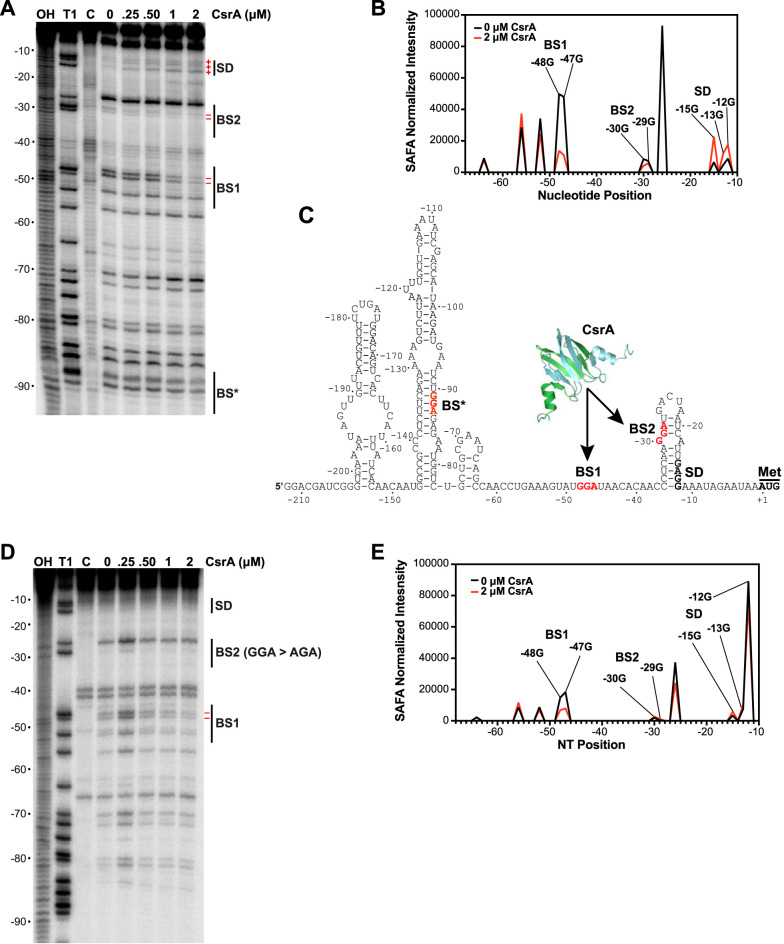
CsrA binding to BS1 and BS2 destabilizes the *ymdA* SD-sequestering hairpin. (A) CsrA-*ymdA* RNA footprint. 5′-end-labeled *ymdA* leader RNA was treated with RNase T1 in the presence of the concentration of CsrA indicated above each lane. Partial alkaline hydrolysis (OH) and T1 digest (T1) ladders are shown alongside a control lane without RNase treatment (control [C]). The position of each binding site (BS1 and BS2), the Shine-Dalgarno (SD) sequence, and a third potential CsrA binding site (BS*) are labeled. Bands showing increased (+) or decreased (–) cleavage in the presence of CsrA are marked to the right of the gel in red. Numbering is with respect to the start of *ymdA* translation. (B) Semiautomated footprinting analysis software (SAFA) quantification of band intensities from panel A. The range of quantification spanned nucleotides −68 to −12. Critical G residues are labeled. (C) Model for CsrA binding to the *ymdA* leader region. GGA motifs of BS*, BS1, and BS2 are in red. The SD sequence and *ymdA* translational start codon (Met) are marked. (D) CsrA-RNA footprint of a transcript containing a mutation in BS2 (GGA to AGA). Labels are the same as described above for panel A. (E) SAFA quantification of band intensities from panel D.

CsrA-*ymdA* RNA footprint experiments were performed to identify CsrA binding sites. RNase T1, which cleaves RNA following single-stranded G residues, was used as the probe. Since we identified four potential CsrA binding sites in the *ymdA* leader region (Fig. 1), all four of the GGA sequences were present in the RNA used in this analysis. Bound CsrA protected the G residues in two GGA motifs from RNase T1 cleavage, indicating that these GGA motifs are components of authentic CsrA binding sites, which we refer to as BS1 and BS2 from hereon (Fig. 3A and B). Cleavage of the G residues in BS2 was much less efficient than in BS1 even in the absence of CsrA (Fig. 3A and B). This observation is consistent with an RNA secondary structure that partially sequesters BS2 in a hairpin (Fig. 3C). We did not observe CsrA-dependent protection of the other two GGA sequences, including the GGA motif that overlaps the *ymdA* SD sequence (Fig. 3A and B). Of particular importance, RNase T1 cleavage of the G residues in the *ymdA* SD sequence actually increased in the presence of bound CsrA. Footprint experiments using a transcript containing a BS2 mutation (GGA to AGA) indicated that CsrA is capable of binding to BS1 in the absence of BS2 (Fig. 3D and E). From these data, we conclude that CsrA binds to BS1 and BS2, which destabilizes the *ymdA* SD-sequestering hairpin.

### CsrA activates *ymdA* translation by promoting 30S ribosomal subunit binding.

**FIG 4:**
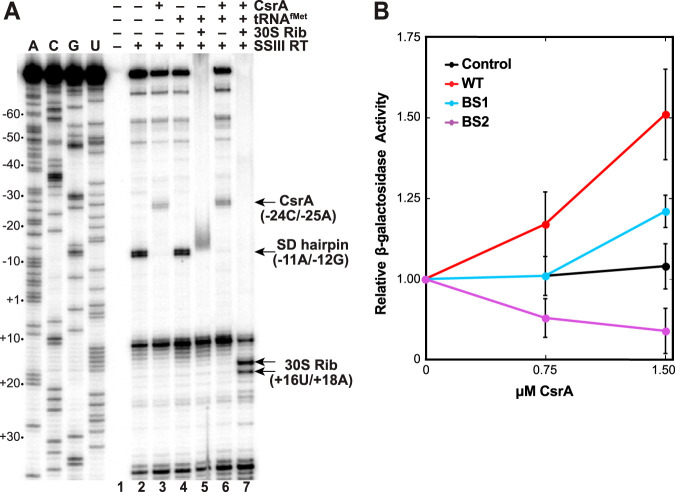
CsrA activates *ymdA* translation by facilitating 30S ribosomal subunit binding. (A) CsrA and 30S ribosome toeprint analysis of *ymdA* leader RNA. The presence of CsrA, tRNA^fMet^, 30S ribosomal subunit (30S Rib), and/or reverse transcriptase (SSIII RT) is indicated above each lane. The positions of CsrA (CsrA), 30S ribosomal subunit (30S Rib), and SD-sequestering hairpin (SD hairpin) toeprints are indicated with arrows. Sequencing lanes A, C, G, and U are marked, and lane numbers are shown at the bottom of the gel. Numbering is with respect to the start of *ymdA* translation. (B) Coupled transcription-translation reactions were performed using the PURExpress kit with wild-type (WT), BS1 mutant (BS1) and BS2 mutant (BS2) DNA templates containing *ymdA'-'lacZ* translational fusions expressed from a T7 promoter. A negative control that was shown previously to be unaffected by CsrA was also used (41). Purified CsrA was added prior to the start of each reaction. β-Galactosidase activity was normalized to 0 μM CsrA for each template. Values are averages ± standard deviations (error bars) from three experiments.

Primer extension inhibition (toeprint) experiments were performed to test whether bound CsrA affects ribosome binding. In this assay, reverse transcriptase will stop just downstream of a bound protein or stable RNA secondary structure. Two bands at positions +12U and +A37 were observed in all experimental lanes (Fig. 4, lanes 2 to 7). RNA structure predictions with Mfold indicate that both of these bands correspond to the base of short RNA hairpins. In the absence of bound CsrA or 30S ribosomal subunits, two adjacent RNA structure-dependent toeprints were identified at positions −11A and −12G (Fig. 4A, lane 2), which are located at the base of the SD-sequestering hairpin (Fig. 3C), indicating that this hairpin forms in the absence of bound CsrA. Two adjacent CsrA-dependent toeprints were observed at nucleotides −24C and −25A (Fig. 4A, lane 3), which is at the 3′ boundary of BS2 (Fig. 3C). To identify the position of bound 30S ribosomal subunit and whether binding was dependent on the presence of CsrA, a ribosome toeprint was also performed. In the absence of CsrA, a smear of reverse transcriptase stops was identified along the 3′ stem of the SD-sequestering hairpin (Fig. 4A, lane 5). This could be a consequence of the 30S ribosomal subunit attempting to access the sequestered SD sequence but failing to make the sequence-specific contacts required for productive binding. Importantly, CsrA-dependent 30S ribosomal subunit toeprints were observed at positions 16U and 18A; the toeprint at 16U is the expected position 15 nt downstream from A of the *ymdA* start codon (Fig. 4A, compare lanes 5 to 7) (19–21). These results indicate that bound CsrA promotes 30S ribosomal subunit binding by destabilizing the *ymdA* SD-sequestering hairpin. One intriguing result that we cannot explain is the absence of bands beyond +12U in the lane containing bound ribosomes (Fig. 4, lane 7). Since the +12U band corresponds to transcripts that did not have a bound 30S ribosomal subunit, we would have expected to see all of the longer bands observed in the control lane (Fig. 4, lane 2).

We next utilized the *in vitro* coupled transcription-translation PURExpress system to determine whether CsrA activates *ymdA* translation. Three different plasmids carrying a *ymdA'-'lacZ* translational fusion driven by identical T7 RNA polymerase (RNAP) promoters were used in this analysis. One plasmid contained the WT *ymdA* leader sequence, while the other two contained a mutation in BS1 (GGA to AGA) or BS2 (GGA to AGA). The expression level of the WT fusion increased with increasing CsrA concentrations (Fig. 4B). The BS2 mutation eliminated CsrA-dependent activation, whereas the BS1 mutation had an intermediate effect. We conclude that BS1 and BS2 are critical for CsrA-dependent activation of *ymdA* translation *in vitro.*

### CsrA activates *ymdA* expression posttranscriptionally *in vivo*.

**FIG 5:**
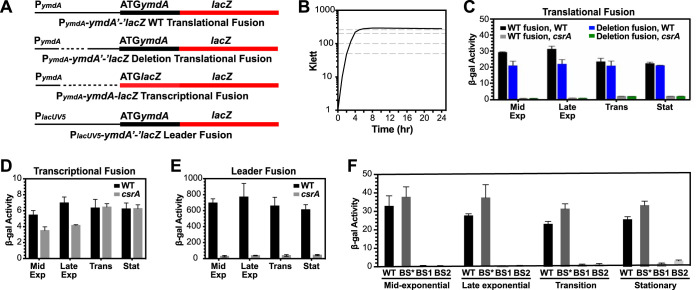
CsrA activates *ymdA* expression posttranscriptionally. (A) Schematic representation of the fusions used in this analysis. The *ymdA* and *lacUV5* promoters and the *ymdA* and *lacZ* start codons are indicated. The *ymdA* leader region is depicted by a thin black line, while the *ymdA* and *lacZ* coding sequences are indicated by thick black and red lines, respectively. The dashed line indicates that this portion of the leader region is absent from the fusion. (B) Representative growth curve of the wild-type strain. Cells were harvested at the growth stages indicated by the dashed lines. (C to E) β-Galactosidase activity (units per milligram of protein) was determined throughout growth in WT and *csrA* mutant strains, in mid-exponential phase (Mid Exp), late exponential phase (Late Exp), transition between exponential and stationary phases (Trans), and stationary phase (Stat). (C) Expression of the *ymdA'-'lacZ* translational fusion. (D) Expression of the *ymdA*-*lacZ* transcriptional fusion. (E) Expression of the P*_lacUV5_*-*ymdA'-'lacZ* leader fusion. (F) Expression of the *ymdA'-'lacZ* translational fusion with GGA-to-AGA point mutations in BS1 and BS2 or GGA-to-GAA mutations in BS*.

To determine whether CsrA activates *ymdA* expression *in vivo*, we monitored expression of a chromosomally integrated P*_ymdA_*-*ymdA'-'lacZ* translational fusion from mid-exponential to early stationary-phase growth in WT and CsrA-deficient (*csrA*::*kan*) strains. This fusion contained sequences between −300 to +4 relative to the start of *ymdA* translation. The *csrA*::*kan* allele contains a transposon insertion following the 50th codon of CsrA’s 61-amino-acid coding sequence, resulting in a 62-amino-acid fusion protein that retains ∼12% of the RNA binding affinity of WT CsrA (24). Expression of the WT translational fusion was 10- to 30-fold higher in the WT strain throughout growth, indicating that CsrA activates *ymdA* expression *in vivo* (Fig. 5A to C). Similar experiments were performed with a translational fusion in which the *ymdA* leader sequence from −211 to −132 was deleted such that it contained only the leader region used in our footprint analysis (Fig. 3). With the exception of a small decrease in expression during exponential-phase growth, the deletion fusion exhibited expression characteristics identical to those of the full-length WT translational fusion including 10- to 30-fold higher expression in the WT strain (Fig. 5C). These results indicate that the leader region used for our *in vitro* footprint studies is sufficient for CsrA-dependent activation of *ymdA* expression. When experiments were performed with a P*_ymdA_*-*ymdA-lacZ* transcriptional fusion in which the *ymdA* leader region is absent, expression levels were similar between the WT and *csrA*::*kan* strains, indicating that CsrA-dependent activation is not due to an indirect effect on transcription initiation (Fig. 5A and D). We next tested a P*_lacUV5_*-*ymdA'-'lacZ* leader fusion in which the *ymdA* promoter was replaced with a promoter that is unaffected by CsrA (4). Therefore, any effect of CsrA would occur after transcription initiation. As was observed with the P*_ymdA_*-*ymdA'-'lacZ* translational fusion, expression of the leader fusion was 10- to 20-fold higher in the WT strain throughout growth (Fig. 5A and E). Taken together with our toeprint and *in vitro* translation results, we conclude that CsrA directly activates *ymdA* expression posttranscriptionally.

Since our footprinting and *in vitro* translation results identified BS1 and BS2 as critical CsrA binding sites, we tested the effect of single nucleotide substitutions in BS1 (GGA to AGA) and BS2 (GGA to AGA) in the context of the P*_ymdA_*-*ymdA'-'lacZ* translational fusion. Both of the individual binding site mutations resulted in the complete loss of the CsrA-mediated activation (Fig. 5F). We also tested whether a GGA-to-GAA mutation in the upstream GGA motif (Fig. 1, GGA*) affected *ymdA* expression. This mutation, which is predicted to maintain WT-like RNA secondary structure, had no effect on CsrA-mediated activation, and therefore, this sequence was ruled out as a CsrA binding site (Fig. 3C and 5F).

### CsrA stabilizes the *ymdA* transcript.

**FIG 6:**
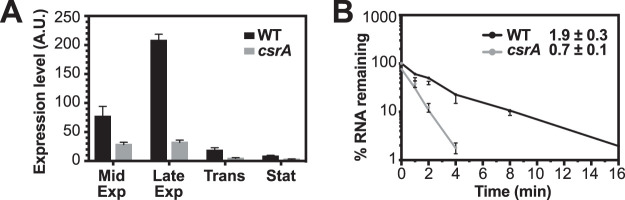
CsrA increases the steady-state *ymdA* RNA levels and the *ymdA* mRNA half-life. (A) *ymdA* mRNA levels throughout growth were determined by qRT-PCR and normalized to 16S rRNA levels. The levels of expression (in arbitrary units [A.U.]) were determined in cells at mid-exponential phase (Mid Exp), late exponential phase (Late Exp), transition between exponential and stationary phases (Trans), and early stationary phase (Stat). Error bars represent the standard deviations of three biological replicates. (B) *ymdA* mRNA half-life in WT and *csrA* mutant strains. Cultures were treated with rifampicin at mid-exponential-phase growth. RNA was isolated at various times after rifampicin addition, and mRNA levels were analyzed by qRT-PCR, and plotted on semilog decay curves. Error bars represent the standard deviations of two experiments with two biological replicates in each experimental set. Calculated mRNA half-lives are shown.

Previous RNA-seq data indicated that *ymdA* RNA levels were 9-fold higher in the WT strain compared to the *csrA*::*kan* strain (6). Since our expression results excluded indirect effects of CsrA on *ymdA* transcription (Fig. 5) and because the stability of a transcript can be influenced by translation efficiency, we performed quantitative reverse transcriptase PCR (qRT-PCR) to analyze *ymdA* mRNA levels in WT and *csrA*::*kan* strains. Depending on the stage of growth, *ymdA* RNA levels were 3- to 7-fold higher in the WT strain (Fig. 6A). Consistent with these results, the mRNA half-life in mid-exponential-phase cultures was threefold greater in the WT strain (Fig. 6B). Whether the stability is a direct effect of CsrA binding or due to CsrA-dependent translational activation was not investigated.

### YmdA represses biofilm formation.

**FIG 7:**
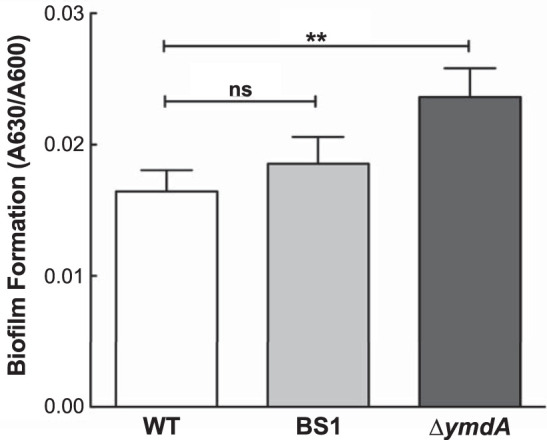
YmdA represses biofilm formation. Biofilm assays were performed in 96-well plates at 37°C in LB medium following the procedure described previously (30). Biofilm formation was quantified as crystal violet staining (*A*_630_) over growth (*A*_600_). Each column represents the average of results from three independent experiments (number of biological replicates *n* = 14 total), with the error bars representing the standard errors of the means and asterisks representing the *P* value (**, *P* = 0.01; ns, not significant). WT, MG1655; BS1, GGA-to-AAG mutation (PLB2839); Δ*ymdA*, unmarked deletion of *ymdA* (AP3080).

A previous study reported that overexpression of *ymdA* inhibited biofilm formation, whereas a *ymdA* knockout strain had no significant effect on biofilm formation (27). Thus, we generated an unmarked deletion of *ymdA* and tested the effect of this mutation on biofilm formation. The Δ*ymdA* strain resulted in a modest 45% increase in biofilm (Fig. 7). Since CsrA activates *ymdA* expression, we reasoned that loss of activation by introducing a BS1 mutation would lead to increased biofilm. Thus, we generated a markerless BS1 mutation using CRISPR. We found that this mutation resulted in a small but reproducible increase in biofilm; however, a Student’s *t* test did not support statistical significance of this effect. Complementation tests of Δ*ymdA* with a plasmid clone of *ymdA* (pYmdA) versus an empty vector control failed to decrease biofilm formation (data not shown).

## DISCUSSION

CsrA is a conserved global regulatory protein that binds to several hundred mRNAs in E. coli (6). CsrA represses translation by a variety of related mechanisms in which CsrA binds near the translation initiation region and/or SD sequence of target transcripts, thereby blocking 30S ribosome binding (1, 2, 19–21). While translational repression is a well-characterized mechanism in bacteria, few examples of translational activation have been reported. Two prior studies provided evidence that CsrA (RsmA) is capable of activating translation, although the underlying molecular mechanisms remain unresolved (25, 26). In this study, we elucidated the first CsrA-mediated translational activation mechanism. CsrA was already shown to activate *flhDC* expression by blocking RNase E access to the transcript (24). In addition, CsrA participates in Rho-dependent transcription attenuation mechanism (22). Thus, CsrA does not function solely as a translational repressor as has been suggested in the literature.

CsrA activates translation of *ymdA* by binding to two sites in the leader region of *ymdA* mRNA, one of which is partially sequestered in an RNA hairpin that also fully sequesters the SD sequence (Fig. 3C). CsrA binding to BS1 and BS2 (Fig. 1 and 3C) destabilizes the *ymdA* SD-sequestering hairpin, leading to translational activation. RNA-seq data in WT and *csrA*::*kan* strains revealed a higher abundance of the *ymdA* transcript in the WT strain (6). We found that CsrA-dependent stabilization of *ymdA* mRNA contributes to that effect.

The function of YmdA is unknown, but a previous study reported that overexpression of *ymdA* inhibited biofilm formation, although a *ymdA* knockout strain had little to no effect on biofilm formation (27). We found that deletion of *ymdA* slightly, but reproducibly, increased biofilm formation (Fig. 7). Although introduction of a BS1 mutation that eliminates CsrA-mediated activation *in vivo* exhibited a small but reproducible increase in biofilm, this difference was not statistically significant. Furthermore, the effect of Δ*ymdA* on biofilm was not complemented in *trans*. Perhaps the effects of *ymdA* and CsrA-mediated activation of *ymdA* on translation would be more substantial under different growth conditions than those used in our studies.

The connection between the E. coli Csr system and inhibition of biofilm formation is well documented (21, 22, 28, 29). CsrA represses biofilm formation by inhibiting synthesis and secretion of the biofilm adhesin molecule poly-β-1,6-*N*-acetyl-d-glucosamine (PGA) (21). CsrA regulates PGA levels by repressing expression of the *pgaABCD* operon, which encodes the cellular machinery required for synthesis, covalent modification, and secretion of PGA (30–32). CsrA represses translation initiation of the *pgaA* transcript by binding to a site overlapping the SD sequence and competing with the 30S ribosome (21). CsrA also participates in Rho-dependent termination of the *pgaA* transcript by destabilizing an RNA secondary structure that sequesters a Rho utilization (*rut*) site, resulting in transcript termination (22). This mechanism provides another example for how CsrA can destabilize RNA structure. CsrA also indirectly represses *pgaABCD* expression by repressing translation of *nhaR*, a transcriptional activator of the *pgaABCD* operon and GGDEF domain proteins that synthesize cyclic-di-GMP, which is the allosteric activator of PGA synthesis (28, 29). Taken together, CsrA-mediated activation of *ymdA* translation might provide another connection between the Csr system and biofilm formation in E. coli, although the weak effects of *ymdA* itself on biofilm under our growth conditions leave this open to question. The location of *ymdA* immediately downstream from the *csgBAC* operon encoding genes involved in the production of curli fimbriae, which mediate an alternative pathway for E. coli biofilm formation (33), allows the possibility that *ymdA* may affect biofilm formation via this alternative pathway.

## MATERIALS AND METHODS

### Bacterial strains and plasmids.

All E. coli strains used in this study are listed in Table 1. The E. coli strain S17-1 λ*pir*^+^ (34) was used for conditional replication, integration, and modular (CRIM)-based plasmid construction (35). The plasmids pLFT, pLFX, and pUV5 (4) were used to construct the translational, transcriptional, and leader fusions, respectively. Plasmid pAR2 contains a P*_ymdA_*-*ymdA'-'lacZ* translational fusion (−300 to +4 relative to the *ymdA* start codon cloned into the PstI and BamHI sites of pLFT). Plasmid pYH370 is identical to pAR2 except that nucleotides −211 to −132 were deleted from the *ymdA* leader region using the QuikChange protocol (Agilent Technologies). Plasmid pAR3 contains a P*_ymdA_*-*ymdA-lacZ* transcriptional fusion (−300 to −221 cloned into the PstI and EcoRI sites of pLFX). Plasmid pAR4 contains a P*_lacUV5_*-*ymdA'-'lacZ* leader fusion (−217 to +4 cloned into the EcoRI and BamHI sites of pUV5) such that the promoter region of *ymdA* was replaced with the *lacUV5* promoter. Plasmid pAR25 contains the P_T7_-*ymdA'-'lacZ* translational fusion (−212 to +4 cloned into the PstI and BamHI sites of pLFT). Mutations in the CsrA binding sites BS1 (GGA to AGA) and BS2 (GGA to AGA) and in the predicted site GGA* (GGA to GAA) in the context of the P*_ymdA_*-*ymdA'-'lacZ* and P_T7_-*ymdA'-'lacZ* translational fusions were introduced using the QuikChange protocol. These plasmids contain a *ymdA'-'lacZ* translational fusion with the *ymdA* promoter or a T7 RNAP promoter driving transcription. WT and mutant fusions were integrated into the chromosomal λ *att* site of E. coli strains CF7789 and TRCF7789 as described previously (35).

**TABLE 1:** E. coli strains used in this study

Strain	Description^*a*^	Source or reference
MG1655	Prototrophic	M. Cashel
TRMG1655	MG1655/*csrA*::*kan* Km^r^	13
S17-1 λ*pir^+^*	*recA thi pro hsdR-M^+^RP4*::*2-Tc*::*Mu* Km::Tn*7 pir*^+^	34
CF7789	MG1655/Δ*lacI-lacZ* (MluI)	M. Cashel
TRCF7789	CF7789 *csrA*::*kan* Km^r^	13
PLB2805	CF7789/P*_ymdA_*-*ymdA'-'lacZ* (−300 to +4) Ap^r^	This study
PLB2806	CF7789/P*_ymdA_*-*ymdA-lacZ* (−300 to −221) Ap^r^	This study
PLB2807	CF7789/P_lacuv5_-*ymdA'-'lacZ* (−217 to +4) Ap^r^	This study
PLB2808	CF7789/*csrA*::*kan* P*_ymdA_*-*ymdA'-'lacZ* (−300 to +4) Ap^r^ Km^r^	This study
PLB2809	CF7789/*csrA*::*kan* P*_ymdA_*-*ymdA-lacZ* (−300 to −221) Ap^r^ Km^r^	This study
PLB2810	CF7789/*csrA*::*kan* P*_lacuv5_*-*ymdA'-'lacZ* (−217 to +4) Ap^r^ Km^r^	This study
PLB2821	CF7789/ P*_ymdA_*-*ymdA'-'lacZ* (−300 to +4, BS2 GGA to AGA) Ap^r^	This study
PLB2822	CF7789/*csrA*::*kan* P*_ymdA_*-*ymdA'-'lacZ* (−300 to +4, BS2 GGA to AGA) Ap^r^ Km^r^	This study
PLB2827	CF7789/P*_ymdA_*-*ymdA'-'lacZ* (−300 to +4, BS1 GGA to AGA, BS2 GGA to AGA) Ap^r^	This study
PLB2828	CF7789/*csrA*::*kan* P*_ymdA_*-*ymdA'-'lacZ* (−300 to +4, BS1 GGA to AGA, BS2 GGA to AGA) Ap^r^ Km^r^	This study
PLB2829	CF7789/P*_ymdA_*-*ymdA'-'lacZ* (−300 to +4, BS* GGA to GAA) Ap^r^	This study
PLB2830	CF7789/*csrA*::*kan* P*_ymdA_*-*ymdA'-'lacZ* (−300 to +4, BS* GGA to GAA) Ap^r^ Km^r^	This study
PLB2831	CF7789/P*_ymdA_*-*ymdA'-'lacZ* (−300 to +4, BS1 GGA to AGA) Ap^r^	This study
PLB2832	CF7789/*csrA*::*kan* P*_ymdA_*-*ymdA'-'lacZ* (−300 to +4, BS1 GGA to AGA) Ap^r^ Km^r^	This study
PLB2839	MG1655/(BS1 GGA to AAG)	This study
PLB3132	CF7789/P*_ymdA_*-*ymdA'-'lacZ* (−300 to −212, −131 to +4) Ap^r^	This study
PLB3133	CF7789/*csrA*::*kan* P*_ymdA_*-*ymdA'-'lacZ* (−300 to −212, −131 to +4) Ap^r^ Km^r^	This study
AP3080	MG1655/Δ*ymdA*	This study

a Numbers in parentheses indicate the cloned *ymdA* region relative to the start of translation, as well as *ymdA* leader mutations. Ap^r^, ampicillin resistant; Km^r^, kanamycin resistant.

Strain PLB2839 contains a scarless GGA-to-AAG mutation in BS1. This mutation was engineered using the no-SCAR (scarless Cas9-assisted recombineering) system described previously (36). The correct mutation was confirmed by DNA sequencing. Strain AP3080 contains an unmarked *ymdA* deletion. This strain was constructed by first replacing the wild-type *ymdA* coding region with a kanamycin resistance gene using λ Red recombinase as described previously (37). The antibiotic resistance gene was subsequently removed using Flp recombinase (38), and the *ymdA* deletion was confirmed by PCR.

### β-Galactosidase assay.

Bacterial cultures containing *lacZ* fusions were grown at 37°C in Luria-Bertani (LB) broth supplemented with 100 μg/ml ampicillin and 50 μg/ml kanamycin for *csrA*::*kan* strains. Cells were harvested at various points throughout growth. β-Galactosidase activity was measured as described previously (19).

### Gel mobility shift assay.

Quantitative gel mobility shift assays followed a published procedure (19). His-tagged CsrA (CsrA-H_6_) was purified as described previously (39). WT and mutant RNAs (−100 to +1 relative to the *ymdA* start codon) were synthesized with the RNAMaxx kit (Agilent Technologies) using PCR-generated DNA templates. Gel-purified RNA was dephosphorylated and then 5′ end labeled using T4 polynucleotide kinase (New England Biolabs) and [γ-^32^P]ATP. Labeled RNAs were renatured by heating for 1 min at 90°C followed by slow cooling to room temperature. Binding reaction mixtures (10 μl) contained 0.1 nM labeled RNA, 10 mM Tris-HCl (pH 7.5), 10 mM MgCl_2_, 100 mM KCl, 40 ng of yeast tRNA, 7.5% glycerol, 0.1 mg/ml xylene cyanol, and various concentrations of purified CsrA-H_6_. Reaction mixtures were incubated for 30 min at 37°C to allow CsrA-RNA complex formation, and then samples were fractionated on a 10% nondenaturing polyacrylamide gel. Free and bound RNA species were visualized with a Typhoon 8600 variable-mode phosphorimager (GE Healthcare Life Sciences). CsrA-RNA interactions were quantified as described previously (19).

### Footprint assay.

CsrA-*ymdA* RNA footprint assays followed a published procedure (19). WT and BS2 mutant (GGA to AGA) labeled *ymdA* RNA (nt −127 to +1 relative to the *ymdA* start codon) was labeled as described above for the gel mobility shift assay. The reaction mixtures were identical to those in the gel shift assay except that the concentration of labeled RNA was raised to 2 nM, and 1 μg of acetylated bovine serum albumin (BSA) was added to each reaction mixture. Reaction mixtures were incubated for 30 min at 37°C to allow CsrA-RNA complex formation, then RNase T1 (0.02 U) was added, and incubation was continued for 15 min at 37°C. Reactions were stopped by adding 10 μl of gel loading buffer (95% formamide, 0.025% sodium dodecyl sulfate (SDS), 20 mM EDTA, 0.025% bromophenol blue, 0.025% xylene cyanol). Samples were heated for 5 min at 90°C and then fractionated through 6% sequencing gels. Cleavage patterns were examined using a phosphorimager and quantified using semiautomated footprinting analysis software (SAFA) (40).

### Toeprint assay.

Primer extension inhibition (toeprint) assays followed a published procedure (19). Gel-purified *ymdA* RNA (150 nM) extending from −79 to +106 relative to the *ymdA* start codon was hybridized to a 5′-end-labeled DNA oligonucleotide (150 nM) complementary to the 3′ end of the RNA in Tris-EDTA (TE) buffer (pH 8) by heating for 3 min at 85°C followed by a slow cooling to room temperature. Toeprint reaction mixtures (10 μl) contained 2 μl of the hybridization mixture (30 nM final concentration), 375 μM each deoxynucleoside triphosphate (dNTP), 10 mM dithiothreitol, and Superscript III (SSIII) reverse transcriptase buffer. CsrA-His_6_ (1.5 μM), tRNA^fMet^ (10 μM), and/or 30S ribosomal subunits (5 μM) were added as indicated. Prior to addition, 30S ribosomal subunits were activated by incubation for 15 min at 37°C. Mixtures of the hybridization reaction and CsrA were incubated for 20 min at 37°C to allow for CsrA-RNA complex formation. tRNA^fMet^ was then added, and incubation was continued for 5 min at 37°C. 30S ribosomal subunits were then added, and incubation was continued for 10 min at 37°C. SSIII (2 U) was added, and incubation was continued for 15 min at 37°C. Reactions were stopped by the addition of 10 μl of gel loading buffer. Samples were fractionated through standard 6% sequencing gels. Toeprint patterns were visualized with a phosphorimager.

### Coupled transcription-translation assay.

*In vitro* coupled transcription-translation assays were carried out with the PURExpress kit (New England BioLabs). Plasmids contained the P_T7_-*ymdA'-'lacZ* fusion with either the WT *ymdA* sequence or a mutation in BS1 or BS2 (described above). A similar P_T7_-*pnp'-'lacZ* translational fusion was used as a negative control (41). These plasmids were used as the templates for coupled transcription-translation reactions following a published procedure (41). Reaction mixtures containing 20 nM plasmid DNA template and various amounts of His-tagged CsrA were incubated for 2 h at 30°C. β-Galactosidase activity was monitored according to the manufacturer’s instructions.

### mRNA half-life analysis.

E. coli MG1655 (WT) and TRMG1655 (*csrA*::*kan*) were grown in LB at 37°C to exponential phase, diluted to an optical density at 600 nm (OD_600_) of 0.01 in fresh LB, and then grown to mid‐exponential phase (OD_600_ of 0.5). Rifampicin was added to a final concentration of 200 μg/ml to stop transcription initiation. One-milliliter aliquots were removed at 0, 1, 2, 4, 8, and 16 min following rifampicin addition and immediately mixed with 0.125 ml of stop solution (10% phenol−90% ethanol [vol/vol]). Total cellular RNA was isolated using hot phenol-chloroform extraction followed by ethanol precipitation. Genomic DNA was digested by treating 20 μg of nucleic acid with 2 U of Turbo DNase (Thermo Fisher Scientific), and RNA was purified from these reactions with phenol-chloroform extraction followed by ethanol precipitation. The overall integrity of the RNA was assessed by observing rRNA following 2% denaturing formaldehyde agarose gel electrophoresis in the presence of ethidium bromide and imaged using a ChemiDoc XRS+ system (Bio-Rad). *ymdA* mRNA levels were detected by qRT‐PCR and plotted on semilog decay curves using Prism (GraphPad).

### Quantitative reverse transcriptase PCR.

Quantitative reverse transcriptase PCR (qRT-PCR) was conducted using the iTaq Universal SYBR green one-step kit (Bio-Rad) and an iCycler iQ5 real-time PCR detection system (Bio-Rad) according to the manufacturer’s instructions as described previously (42). The primer sequences used for this analysis were 5′-CTCTCTTATGCTCGGCAGTTT-3′ and 5′-ACATGCCGGTTCCACAAT-3′. Reaction mixtures (10 μl) contained 200 ng of RNA or standard DNA, 300 nM each primer, iScript reverse transcriptase, and 1× iTaq Universal SYBR green reaction mix. Reaction mixtures were incubated for 10 min of reverse transcription (RT) at 50°C, 1 min of RT inactivation at 90°C, followed by 45 cycles of denaturation for 10 s at 95°C and annealing/extension for 20 s at 60°C. Following amplification, melting curve analysis was used to verify the specificity of the PCR product according to a single melting temperature (*T_m_*). Melting curve analysis consisted of incubation for 1 min at 95°C, 1 min at 60°C, followed by 70 steps in which the temperature was increased to 95°C at a rate of 0.5°C/10 s/step. The *ymdA* mRNA concentrations were determined relative to a DNA standard curve for the PCR products using iQ5 optical system software version 2.1 (Bio-Rad) and were normalized to 16S rRNA levels.

### Biofilm assay.

Biofilm assays of WT (MG1655) and Δ*ymdA* mutant strains, with or without complementation of *ymdA* via ASKA plasmid clone pYmdA versus the empty vector pCtrl (27), were performed in 96-well plates at 37°C in LB medium following the procedure described previously (30). Growth (*A*_600_) and crystal violet staining (*A*_630_) was measured using a BioTek Synergy HT plate reader, and biofilm formation was quantified as crystal violet staining (*A*_630_) over growth (*A*_600_).
